# Study on landscape evaluation and optimization strategy of Central Park in Qingkou Town

**DOI:** 10.1038/s41598-022-06006-z

**Published:** 2022-02-07

**Authors:** Lingyan Xiang, Yunqing Tian, Yucong Pan

**Affiliations:** 1 Department of Environmental Art Design, Shanghai Academy of Fine Arts, Shanghai University, Shanghai, 200040 China; 2 Department of Culture and Media, Ningde Vocational and Technical College, Ningde, Fujian China

**Keywords:** Sustainability, Urban ecology

## Abstract

This article mainly discusses the evaluation and optimization of the green space utilization value of comprehensive parks used by people in dense urban areas based on the desire for green and healthy living in the postepidemic era. As a qualitative study of urban parks, this study builds an evaluation system based on the American landscape performance series and combines it with comprehensive indicators of China’s urban parks, including environmental performance (such as park planning, infrastructure, trails, and vegetation), health performance (such as cultural education, park activities, and transportation accessibility) economic performance (such as tourist consumption and stimulating the development of surrounding construction) and three other aspects: conducting a site evaluation; evaluating observed behavior, interviews and questionnaires; and performing the analytic hierarchy process–coefficient of variation weight comprehensive evaluation analysis. Additionally, the park comprehensive index, land use index, traffic convenience, park vitality index and other dynamic changes are analyzed over time. The purpose is to explore the foundation of urban parks after the epidemic. The role of the urban park environment in sustainable ecological development is verified, and appropriate optimization and improvement actions are determined.

## Introduction

With the number of confirmed COVID-19 cases and deaths increasing, the outbreak has created an unprecedented health crisis for human society. Residents’ demand for parks and public green spaces has increased significantly since the outbreak, highlighting the important benefits that natural green environments can provide in the face of such epidemics^1,2^. Especially during the global lockdown, people could still use natural green spaces to safely engage in outdoor activities with their families and maintain their physical, mental and social well-being^3^. A growing number of studies have shown that due to demographic, political, economic, cultural and economic factors, parks in developed and developing countries are unevenly distributed^4–6^. Areas where low-income people live have insufficient parks and outdated facilities, while in relatively affluent areas, the opposite is true^7,8^.

In developing countries such as China, with the development of urbanization, the construction of urban green space has steadily improved. In addition to parks, urban green spaces, and botanical gardens, street-corner parks and pocket parks have been constructed and used^9^. However, due to lack of public awareness, poor maintenance and management and lack of adequate infrastructure in poor areas, the parks in small towns have not been fully utilized. Since the epidemic, a large number of studies have considered green space as a primary measure for improving human health^10^. Because the health and well-being benefits of a park depend largely on users’ primary impression, this impression determines whether the park is attractive enough to encourage residents to visit it again^11^. Since the epidemic, due to rapid changes in the global environment, population, and technology, comprehensive performance evaluation and analysis based on the behavior of tourists in parks in small towns as well as site evaluations have become means of quickly confirming the sustainability of parks^12^. The planning and development of green space in parks, especially in urban areas with unevenly distributed green spaces, should follow the principles of sustainable development to improve the quality of life of urban residents^13^. Studies have shown that the relationship between park green space and health is most obvious in groups with low socioeconomic status, such as the populations of towns or rural areas, and urban construction around parks is often better than that in urban fringe areas^14^. However, urban parks and green spaces have not been fully utilized. In addition to the uneven distribution of green spaces, the quality of parks may play an even more critical role in determining the use of park green spaces^15^. Existing research lacks a focus on and evaluation of the design elements, natural elements and architectural facilities of parks in relatively poor areas of developing countries^16,17^. The purpose of this research is to evaluate relatively poor urban comprehensive parks.

With the rapid growth of the construction of small towns, small town parks are facing huge challenges in the construction of the ecological environment. In recent years, China has gradually increased the construction of its green infrastructure, especially after the implementation of the “National New Urbanization Plan (2014–2020)”. Small town parks, which represent a small number of green spaces in cities and towns, actively explore the relationship between park green spaces and the environment and the health of residents through all-around service targets and diversified functions. However, compared with urban comprehensive parks, small town comprehensive parks lag slightly in terms of construction and maintenance (“Trends and Planning Choices after China’s Urbanization Rate of 60%”). Additionally, they lack corresponding guidance. Especially after experiencing the epidemic, the thinking of small town residents has changed, and the problem of inequality of green space exposure has appeared in small towns^18^. Existing small town parks cannot meet the needs of urban green infrastructure and ecological sustainability. Regarding development needs, to make full use of existing park spaces to effectively promote health and human well-being, it is necessary to thoroughly explore how the residents of small towns evaluate parks after using them. These evaluations will help improve the usability of the parks and find a suitable solutions for outdated park facilities^19^.Taking the Central Park of Qingkou Town, Minhou District, Fuzhou City as an example, this article focuses on how to cost-effectively intervene in the conditions of comprehensive parks in small towns and proposes corresponding optimization suggestions for improving urban green public spaces and park designs. The research refers to LSP and divides park benefits into three categories^20^: environmental performance (such as park planning, infrastructure, healthy trails, and vegetation), health performance (such as cultural education, park activities, and traffic accessibility), and economic performance (such as tourist consumption and stimulating the development of surrounding construction). In addition, other factors are considered that affect the behavior of tourists in the space and the interest in and popularity of the park so that these factors can be adjusted and enhanced to benefit the activities of tourists (such as walking, jogging, and picnics) or improve mental health (such as reducing stress and depression).

### Overview of the site

**Figure 1:**
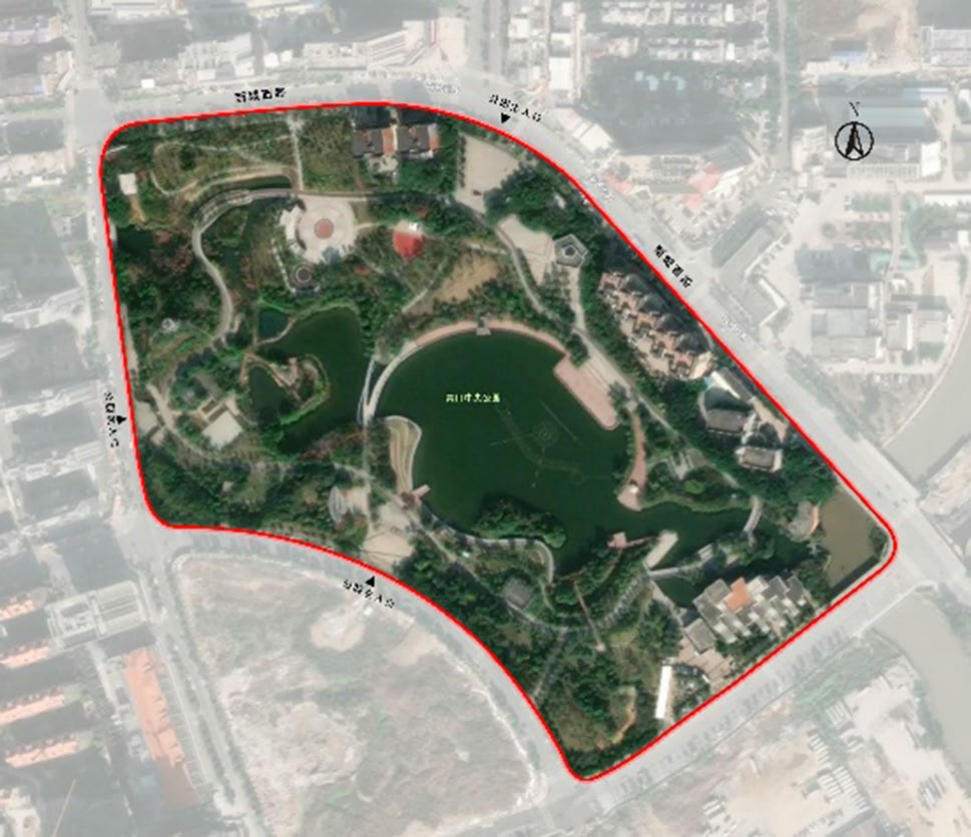
Schematic diagram of the Qingkou Central Park research area.

Qingkou Town is located in Minhou District, Fuzhou City, Fujian Province. The current population is 85,865 (2020). In the past ten years, this town has increased by only about 9% with 4377 additional people (population 81,488 in 2010). The low growth rate is due mainly to the closeness of the town and the city, which allows a large number of people to migrate to the city. The urban pattern of Qingkou Town has changed rapidly over the past ten years. For example, in June 2002, Qingkou Investment Zone became a Fuzhou City-level zone dominated by the automobile industry. In the industrial zone, with the continuous advancement of the industry, in 2012, Qingkou Town was listed in the third batch of national development and reform pilot towns (announced in the launch of the supplement and adjustment work of national key towns); at the same time, Qingkou Town was positioned as part of Fuzhou: a livable city with a beautiful environment at the southern gate of the city and became the country’s first new modern automobile city. To improve the inequality of green space in parks, the construction of Central Park in Qingkou Town began in August 2012 (Fig. 1), and the park was opened to the public in early 2015. Qingkou Central Park is located in the center of Qingkou Town. It is an open comprehensive park with water, leisure, entertainment and ecology together serving the purpose of improving the ecological environment. The total area of the park is approximately 138,000 square meters, including 40,000 square meters of water and 65,000 square meters of green area. Located at the confluence of the Minjiang River, Wulong River and Majiang River, the park has a good natural environment and a humid climate. The surrounding vegetation is abundant, and there are many streams. The water body inside the park is artificially excavated and filled with water from the surrounding streams. The original intention of the design was to take the natural ecology of the area as the design concept. The set view, tour, popularization of science, and ecology all serve the public place. Due to the poor effect after the completion of the park, the flow of people has gradually declined, public satisfaction is not high, facilities and equipment are not complete and other prominent problems have arisen. To a large extent, these factors affect tourist visitation behaviors and psychological feelings. Therefore, it is universal to study Qingkou Central Park as a typical small-town park.

## Research methods

Comprehensive parks in small towns generally serve the urban residents within a few kilometers of the park. Parks are generally in an area with a large flow of people in a small town^15^. However, the geographical blockage indicates that the users of parks in small towns are generally of limited educational level, so the investigation process is more complicated. This study adopts a multimethod design with DPOE (diagnostic post-use evaluation) as the main research method, supplemented by the analytic hierarchy process (AHP) and GIS technology, to quantitatively evaluate the current use of comprehensive parks in small cities and towns, and make a decision based on the evaluation results regarding the corresponding optimization and promotion strategy. The research was divided into the following three stages.

### Evaluate

#### Basis of evaluation

Based on the perspective of “human body—movement—space—place—environment”^21^, stimulation theory and control theory in environmental psychology are used as the main directions for setting up and investigating recreational behaviors. Field investigations were conducted on the current environment, region, culture and other relevant factors of Central Park in Qingkou Town from April to May 2020. The survey covered weekdays, weekends and holidays, sunny/rainy days and mornings and evenings. The main task of the survey was to supervise the development and use of parks in small towns, the number of users, the types of facilities, and the appearance and maintenance of the park^22^ and to list the problems related to the environment and its ecology, the benefits provided by groups of parks, or the benefits provided to the surrounding enterprises and schools. Especially after the epidemic, residents have had a more active and urgent need for the participation of green space. To cover a wider range of weather and time conditions, data were collected in sequence during the observation period according to a preset scheme for four periods of the day, alternating between two working days and one weekend each week^23^. Two team members (interviewers) were in contact with tourists at different times in the park from 8 am to 8 pm. In order to minimize selection errors, each respondent was invited to participate in the survey (the targets included adults, children, and adolescents). Elderly people who agreed to participate in the survey were asked about their visit activities and usage behaviors in the park, such as the frequency of visits, distance, and satisfaction with respect to park maintenance, safety, and infrastructure construction^24^. Finally, the survey requested relevant social demographic information, such as age, highest education level, and marital status (with or without children).

#### Construction of a performance evaluation index system for comprehensive park landscapes in small towns

The evaluation index of the general applicability of small-town parks was established using a field investigation and the combination of the American landscape performance series (LPS)^25^. The index system was divided into three levels. The first level was the target level, that is, the evaluation index system of the comprehensive landscape performance of small-town parks. The second level was the criterion level, including environmental performance, health performance and economic performance. As the embodiment of the second level, the three-level index layer mainly includes park construction, infrastructure setting, landscape quality, garden atmosphere, and tourist behavior. Considering the accuracy of comprehensive park evaluation in small towns, the three-level index layer was used to obtain 19 related indexes after expert advice and screening^20^.

#### Questionnaire design

Since this study is aimed at parks in small towns and the surrounding residents generally have a low level of education, to obtain more effective data, the questionnaire was in the form of ticking. In terms of content, the questionnaire was divided into two parts. The first part collected basic information about tourists, such as the mode of transportation to the park and visit frequency. Second, there were 19 evaluation index factors. The Likert method was used to evaluate the index^26^, and the answers each had five levels: very satisfied, satisfied, average, dissatisfied and very dissatisfied.

#### Combination weight analysis

Some papers in the past ten years have discussed the limitations of the AHP in dealing with the complexity and uncertainty of evaluation indicators and used fuzzy comprehensive evaluation methods to deal with the problem of uncertainty^27–30^. However, the fuzzy evaluation method has gradually been eliminated due to its inability to quickly determine the evaluation content^31^. Therefore, this study used indicator weights for analysis and evaluation. On the basis of the AHP analytic hierarchy process, the coefficient of variation (CV) is added to the weight of each indicator^32^. The main purpose of adopting this method is to establish a landscape performance evaluation system for Qingkou Central Park, refer to the satisfaction evaluation of tourists through the DPOE, and determine the content that the park needs to be optimized.

### Survey

The places where tourists gather or where tourists are the most often have a certain reference significance for the planning and design of parks. Therefore, collecting data on tourist gathering places is needed in the research process^33^. An increasing number of studies have pointed to the use of social media to examine users’ daily life behaviors and spatial distribution relationships. Wood et al. attempted to evaluate the access rate of entertainment venues based on the location of photos posted on Flickr^34^. Hamstead et al. use geolocation data from Flickr and Twitter to assess changes in the use of all parks in New York city^35^. Because the identification system for comprehensive parks in most small towns is not clear, tourists cannot clearly identify a place to visit or a location they often visit. Therefore, the following attempts were made: (1) A total of 182 points of interest of tourists in Qingkou Central Park were collected through Octopus Aata Collector 8, Six-Feet and other software. (2) The distribution of interest points and on-site observations were used to identify five gathering points that cover most of the park landscape. They were named A, B, C, D and E (Table 1) for fixed video recording. The average weekday traffic and weekend traffic information was obtained. (3) The collected data were imported into Excel for sorting, and the utilization of parks was visually expressed in different time periods through GIS. (4) From May to August 2020, 300 copies of paper questionnaires were distributed at nodes A, B, C, D and E. (5) In addition, the questionnaire content was imported into Excel for simple processing, classification and deletion of duplicate data. (6) Finally, the spatial and temporal distribution results and comprehensive evaluation results of tourists in comprehensive parks in small towns were obtained, and optimization suggestions were summarized.

**Table 1:** Landscape node and application of the Central Park.

Node name	Category	Radiation function	Theme settings	Application status
Node A	Northeast main entrance square	Square, Sunshine Lawn, Culture Stone, Tree Array, Running Road (Park Road)	Evacuation, leisure, recreation, running, sports, picnics, reading, etc	The stream of people gathered in the sunny lawn with a slight slope. In contrast, the shading area of cultural stone and tree array is mainly the sports area used for children’s climbing and badminton. The running track is the area with the strongest personnel mobility. Most of them are young people running and middle-aged and old people walking
Node B	Southwest main entrance square	Square, lawn under the forest, walkway around the lake, landscape bridge	Camping, picnic, evacuation, running, parent–child leisure, etc	The understory lawn area is the most active area in Central Park. Visitors will have picnics, watch the lake, and rest in hammocks (by trees no smaller than 15 cm in diameter). In addition, the space where children are most active is the area used for skateboarding with the bridge slope near the lawn area under the forest. In general, the playing heat of node B is high. The age distribution of the population is relatively balanced
Node C	West secondary entrance square	Badminton court, ecological tower, ecological wetland	Evacuation, leisure, health for the elderly, ecological wetland sightseeing, natural science popularization, etc	The road confluence area on the west side is dominated by the ecological wetland surrounding the sports ground. At present, the main activities are preschool children’s entertainment, and teenagers’ viewing of wetland fish ponds. Compared with other areas, the function settings are not clear enough, and the flow of people is rarely concentrated, with strong mobility
Node D	Sports Theme Square	Sports Square, Skateboard Square, Fitness Square	Square, fitness and entertainment, all-age health and fitness	In addition to a small number of people in the area with facilities in the sports square, other areas are relatively empty with imperfect functional facilities. The D node area is far from other areas, so scattered tourists will choose to squat (lack of seats) on the pavement around the square for a short rest
Node E	Waterfront wooden platform	Viewing wooden platform, leisure pavilion	Lake view, overlooking, leisure and entertainment	Since the waterfront wooden platform is fully illuminated from 09:00 to 16:00, although it is mainly an open viewing space, tourists will not stay in the absence of shelter. Compared with the leisure pavilion on the opposite side of the lake and the waterfront lawn revetment as a relatively private area, there will be a certain number of tourists who stay and have a rest

#### Spatial distribution and experience of respondents

The behavior track, gathering area and spatial distribution of tourists in the park affect the evaluation of the park. However, due to the different behavioral habits of tourists, some areas of the park will have a crowd gathering effect^36^. As a result, the use of environmental resources in the park is uneven, resulting in the waste of environmental resources and ecological damage.

**Figure 2:**
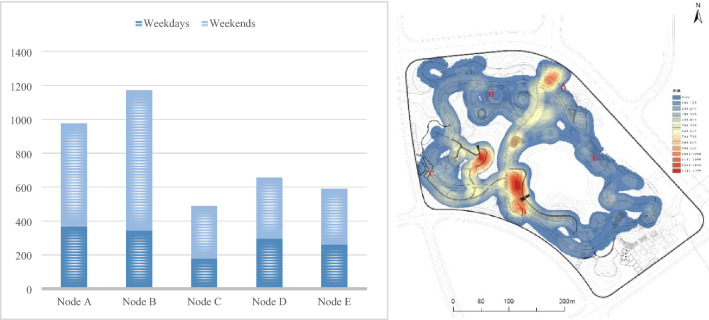
Schematic diagram of the flow of people in different times and spaces.

During the study, to make the data more accurate, a total of six samples were randomly selected from three working days and three weekends from May to July 2020 for data collection. The collection method consisted of five people at five important nodes in the park that basically cover the popular areas (including A. Northeast Main Entrance Square, B. Southwest Main Entrance Square, C. West Secondary Entrance Square, D. Sports Theme Square, and E. Waterfront wooden platform); the sites were filmed from 06:00 to 20:00 (fifteen minutes were taken from 06:00–08:00, 08:00–11:00, 11:00–13:00, 13:00–17:00 and 17:00–20:00 for each time period) to record the activity track of tourists in a day. Then, the average weekday samples and weekend samples were averaged, and the data of the flow of people and the length of stay were processed. The flow of people in the different periods of each node of the two samples was sorted using Excel, and coordinate marks were made on the construction drawings according to the image data to screen out repeated and unreasonable data^37,38^. Then, ArcGIS was used to perform data visualization (scenic spot heat), and the following conclusions could be drawn: there was a significant difference in the stay time of tourists at different nodes (Fig. 2).

Through node analysis, it was found that tourists stayed in the park for at least 30 min. Compared with the research process, it took approximately 1 h and 10 min to complete the whole track through the Six-Foot app, which showed that tourists stayed in the park for a longer time. Among them, the passenger flow peaks were during 06:00–08:00, 11:00–13:00 and 17:00–20:00, while the passenger flow was lower at other times. In addition, according to observation and survey data summary, passenger flow was not evenly concentrated among the five node areas^6^. Overall, node A and node B had a large flow of tourists and a longer stay time. Node D was ranked next, while node C and node E had less traffic and shorter stay times.

#### Overview of respondents

According to the POE field survey and questionnaire survey, 292 valid questionnaires (recovery rate 97.3%) were obtained, among which 58.2% (169 persons) were female, slightly higher than the 41.8% (123 persons) that were male. Among them, 40.5% (118 persons) were carrying children under 8 years old. In terms of age, young people between 30 and 39 years old accounted for 43.7% (128 people), most of whom carried children, followed by younger people between 19 and 29 years old and young people between 40 and 49 years old, which fully showed that the urban center where the park is located was dominated by young people. Among the visitors, 71.9% (210 people) were local residents, 18.5% (54 people) were temporary residents, and 9.6% (28 people) were foreign tourists. In terms of how people arrived at the park, most of them came by walking (116 people), accounting for 38.9%, followed by electric vehicles and private cars (61 people each), accounting for 21.6%. Among them, 53% (154 people) chose to come to the park when they were free, and 24% (70 people) came to the park three or four times a week. In the park system of this small town, the public green space served the local residents to a large extent and gradually became a recreational place for the real-time entertainment of nearby residents (Table 2).

### AHP-CV comprehensive weight analysis

The development of the AHP-CV combined weights has led to a change in the method of determining indicator weights from a single subjectiveness to a comprehensive objectivity^39^. In order to avoid the evolution of the AHP to a single weighting method, the AHP method is combined with the SW and CV methods here to calculate objective weight, based on the principle of minimum information entropy combining two kinds of weighted information^40^.

1. To ensure the reliability of the data, first, check the consistency of the paired comparison matrix:
  $${\text{RC }} = {\text{ IC}}/{\text{IR}}$$
  If RC < 0.1, the result is acceptable and has the significance of in-depth research.
2. The CV method of calculating objective weights is a statistical measure of the degree of dispersion of the probability distribution or frequency distribution^32^, that is, the ratio of the standard deviation to the evaluation mean, expressed as a certain degree of change. This study uses the CV method to calculate objective weights.
  Coefficient of variation calculation model:
  $$C_{i} = \frac{{\sigma_{i} }}{{\mu_{i} }}$$
  Weight calculation model:
  $$W_{2i} = \frac{{C_{i} }}{{\mathop \sum \nolimits_{i = 1}^{n} C_{i} }}$$
3. Combine the obtained weight i with the weight obtained by CV, and the combined weight of each evaluation index adopts the calculation principle of minimum information entropy. The combination of methods makes full use of the AHP and CV methods and solves the uncertainty problem caused by the fuzzy analysis method.

Method model:

$$W_{i} = \frac{{\sqrt {W_{1i} W_{2i} } }}{{\mathop \sum \nolimits_{i = 1}^{n} \sqrt {W_{1i} W_{2i} } }}$$

### Experiments statement

All test methods were performed in accordance with relevant guidelines and regulations. All pilot schemes have been approved by the ethics committee of Fuzhou Municipal government, including any relevant details. Informed consent has been obtained from all participants in the questionnaire and/or their legal guardians (Tables 2, 3).

**Table 2:** Characteristics of visitors.

Demographics	n	Percentage
**Gender**		
Male	123	41.8%
Female	169	58.2
**Age**		
Above 18	21	7.2%
19–29	83	28.3
30–39	128	43.7
40–49	32	10.9
50–59	15	5.1
Over 60	14	4.8
**Resident**		
Yes	210	71.9%
No	92	29.1
**Education**		
High school and below	200	68.3%
Junior college	53	18.1
Undergraduate	36	12.3
Postgraduate and above	4	1.4

**Table 3:** Qingkou central park evaluation index weight and weight ranking.

Code	Evaluation Factor	AHP Weight	Ranking	CV Weight	Ranking	Combined Weight	Ranking
Environmental performance A	Park planning A1	0.082	3	0.05	11	0.068	2
	Landscape composition A2	0.039	13	0.047	13	0.045	14
	Number and quality of facilities A3	0.074	5	0.032	17	0.052	9
	Plant diversity A4	0.046	11	0.061	6	0.056	8
	Water environment comfort level A5	0.031	15	0.062	5	0.046	13
	Running track comfort A6	0.022	19	0.06	7	0.038	19
Social performance B	Convenience of participation by the disabled and the elderly A7	0.042	12	0.05	10	0.048	12
	Coordination with surrounding environment A8	0.053	7	0.044	15	0.051	10
	Scenery quality B1	0.108	1	0.028	18	0.058	6
	Science education value B2	0.03	16	0.071	3	0.049	11
	Historical and cultural values reflected B3	0.049	10	0.059	8	0.057	7
	Industrial cultural value embodiment B4	0.037	14	0.043	16	0.042	17
Economic performance C	Location recognizability B5	0.08	4	0.048	12	0.065	3
	Traffic convenience B6	0.093	2	0.052	9	0.073	1
	Health index B7	0.067	6	0.046	14	0.058	5
	Catering facilities C1	0.024	17	0.072	2	0.043	16
	Tourist consumption C2	0.023	18	0.076	1	0.044	15
	Well-being C3	0.05	9	0.027	19	0.039	18
	Stimulate the development of surrounding construction C4	0.051	8	0.066	4	0.061	4

## Results

The survey results show that low-income and poorly educated youths rely more on parks and green spaces and use them frequently^41^. For low- and middle-income people, parks are the most convenient way to experience nature and enjoy leisure. The most economical way to practice such pursuits is to visit a park^42^. Most visitors to Qingkou Central Park are local residents, and visitors who are nearby often come to the park to relax. Visitors to the park choose their resting place based mainly on sun exposure (shady location)^43^. When tourists were asked about optimization suggestions for the park, they provided a variety of answers, the most common of which was the hope that a children’s play area could be established to distract children from adult activities.

When the results of the questionnaire survey and the analysis of the classification of interaction patterns between space and behavior are combined^44^, the results indicate that pure green space cannot effectively attract people. The in-depth analysis, combined with the video records of the five crowd gathering points A, B, C, D and E, showed that tourists stay in the park for at least 30 min during holidays and peaceful times. Young and middle-aged freelancer workers or individual practitioners and their children have more time and opportunities to relax in the park, and their length of stay in the park is relatively free. The interviews revealed that based on the differences in the cultural level of the urban population, Qingkou Central Park is less convenient for some visitors. For some green leisure parks, the respondents’ evaluation of environmental performance A for carrying park infrastructure is low, their evaluation of economic performance C is relatively high, and they place health performance B second. The highest comprehensive weight evaluation is traffic convenience B6 (0.073). These findings indicate that the park site needs to consider traffic convenience, followed by park planning A1, which illustrates the importance of the overall planning of the park to the construction of small town parks. The design of Qingkou Town’s Central Park, which divides the road surface of the park into pathways and amusement areas, meets the needs of more fitness enthusiasts. The comprehensive weight shows that the location recognizability B5 (0.065) stimulates the development of surrounding construction, and C4 (0.061) receives a high evaluation. Thus, the park basically meets the needs of tourists.

The lower score in environmental performance A is because of the convenience of participation by the disabled and the elderly A7 (0.048). Qingkou Central Park has a wide range of services and a strong comprehensiveness, but there is almost no design for access for people with disabilities, including the large-scale use of squares, glossy marble, barriers at entrances and exits, etc. Interviews with middle-aged and elderly participants, especially those over 65, revealed that they generally choose to visit the park at night (18:00–21:00) and mainly occupy the square in the park for group activities such as square dancing. They seldom enter the park during the day, and the low light at night greatly increases the difficulty of the elderly in entering and exiting the park in rainy conditions or when there are other barriers. Secondly, the landscape composition A2 (0.045) and the coordinated development with the surrounding environment A8 (0.051) were evaluated as fair. In the survey, the interviewees generally agreed that the park lacks rigid infrastructure, such as rest facilities and leisure seats. From the point of view of the three crowds of A, B and C, the places where the crowds rest are mostly lawns or places where they can sit on the ground. Some interviewees reported that Qingkou Central Park is not as fun as the parks in downtown Fuzhou. Many tourists come from small places but, affected by the network and the external environment, are looking forward to the construction of small town parks and even hope to obtain the same experience as the infrastructure and environmental construction of parks in large big cities.

In terms of health performance level B, although Qingkou Central Park is located in the center of Qingkou Town, the respondents gave high scores to traffic convenience B6 (0.073) and location recognizability B5 (0.065), but the traffic in the park was rated as very important for the first time. Those who are not familiar with the park are unsure of the flow of the layout. According to the 129 data lines uploaded by crawling tourists, 65% of the data shows that the traffic routes in the park are not smooth enough, and the migration routes are relatively slow. Additionally, 23% of the data show that tourists did not visit the entire park but only walked through half or 1/3 of the area before ending their visit; the traffic outside the park shows that for those living in the town, the park is slightly more remote, that is, more than 20 min’ walk. For tourists with electric cars or private cars, a trip of more than ten minutes is not attractive enough; thus, this group visits less frequently, and more people think that it takes more time (about 30 min by car) to go further for a more interesting experience. The evaluations of historical and cultural value reflected in B3 (0.057) and industrial cultural value reflected in B4 (0.042) show that the interviewees had a low sense of identification with the regional culture conveyed by the park. From the perspective of the importance of the system, historical and cultural values ​​and industrial cultural values need to occupy a higher proportion in the planning and design of the park, especially because Qingkou Town has a long history and culture and at the same time, Fuzhou is a key automobile industry base. The medium-bearing regional culture makes small towns more recognizable and at the same time plays the role of cultural publicity and promotion. The index factor in economic performance C is relatively high, and the lowest evaluation is that of the catering facility C1 (0.043). Most interviewees thought that the catering facilities in the park were insufficient or unreasonable; for example, some catering facilities are exposed to the scorching sun or located on sections of roads with frequent traffic, resulting in a poor dining environment (Table 3).

## Discussion

### General discussion

In recent years, small-town industries have faced transformation and upgrading, which has encouraged more young people to find employment in small towns. Parks and green spaces in small towns indirectly affect the livability of towns and the satisfaction of small-town residents. The original living habits of the people who use the park may affect the utilization rate of the park, especially in small towns where green spaces or other outdoor entertainment venues are scarce^45^. On the basis of beautifying the environment, parks and green spaces in small towns built in the right place should guide a healthier lifestyle^46^. As a comprehensive park in a small town, Qingkou Central Park has insufficient infrastructure, a lack of landscape functions and monotonous landscape elements, which do not fully reflect the value of the park. This phenomenon is common in small town parks in China.

This research mainly used a POE field survey and ArcGIS^38^ flow heat map to study the usage of Qingkou Central Park, and the specific objectives were as follows. (1) Qingkou Central Park draws on the design concept of New York’s Central Park in the planning, including natural layout, open space, large-scale use of lawns, etc., but ignores the basic principles of adapting measures to local conditions. The entire park lacks the necessary infrastructure (such as seats, trash cans, kiosks, and toilets). Tourists tend to rest on grass or lawns under trees, resulting in exposed soil on some lawns, indicating that the tourist capacity has exceeded the environmental load capacity, especially at the waterfront lawn at node B, because it is close to the only toilet in the park and close to the entrance and exit. This good location attracts a dense crowd. (2) The lack of landscape function is another problem faced by comprehensive parks in small towns, and this problem is mainly manifested in several aspects. First, the number of children suddenly increased after China opened up its second-child policy. There is no special playground for children in the park. As a result, children in Qingkou Central Park are scattered throughout the park, which increases the risk factor^47^. Second, although it is an open park, all entrances and exits of the park are S-shaped and semi-blocked, and individual entrances and exits are in the form of steps. It is difficult for the elderly or disabled with mobility issues to participate in park activities. Third, node C is the ecological area of the park, and park roads are unreasonably planned, which makes it difficult for tourists to enter. During the investigation, it was found that a large area of idle grass was covered with spider webs^48^. Due to inadequate management and control, some waters were messy, and rainy weather causes it to emit a peculiar smell, which strongly affects the ecological balance. (3) In the interviews, most of the tourists thought that the corresponding cultural elements should be added to facilitate the guidance of children to understand the local historical and cultural characteristics. Compared with the automobile-led industrial culture in Qingkou Town, only 17% of the people thought that elements of automobile culture could be added to the park. This phenomenon showed that tourists had a lower acceptance of local industrial culture and a higher recognition of historical culture^49^.

### Based on the above content, the optimization strategy of Qingkou Central Park is analyzed as follows

#### The rationality of the park’s landscape design, leisure activity area and playground design will affect the activities of park users

A mobile toilet should be added to the location to increase tourists’ enthusiasm for visiting^50^. Approximately 40.5% (118 people) will visit the park with children under the age of 8, as a common reason for visiting the park was “bringing children”. Therefore, the design and venue of the children’s amusement area will be helpful in improving the utilization rate of the park^51^. The current guidance system is inadequate, and the style is not uniform. It is recommended that a visual guidance system be set up at entrances and exits, squares, road intersections, etc. During the optimization process, the visual guidance system should be combined with the publicity column and the background of the park. The column of cultural celebrities is uniformly designed to enhance the recognizability and orientation of the park.

#### The rest space of the park should be pleasant and accessible

Visitors can enjoy a healthy and beneficial environment in such a space and simultaneously offset the depression and exhaustion of urban environmental life. Through a combination of field surveys and questionnaires, the impact of urban green space as a learning environment is investigated. These impacts are concentrated mainly on children. Parks should add special learning activity spaces for children and teenagers to form a crowd diversion; the research design shows that natural areas can help children to concentrate, relieve stress and relax^52^. Therefore, parks should renew some of the unused squares and add cultural elements to them to guide visitors to sense their inheritance of the humanities. Additionally, flowering trees and shrubs should be properly planted in all nodes of the park, as the color and fragrance of plants can relieve monotony The park atmosphere can enrich the learning and work functions of the park green spaces and play a role in regulating physical and mental health.

#### Researchers have pointed out that accessibility is a key factor when designing public parks

Therefore, the addition of park roads should be considered for the C node and its surrounding wetland system^53^. It is recommended that native fast-growing shade trees be planted in the large area of lawn near the wetland to disperse the picnic crowds at nodes A and B and to meet the needs of the surrounding sports venues and open-air squares. The rest of the area combines artificial and wilderness landscapes, which not only meet the needs of amusement areas but also ensure ecological balance; research has found that nearly a quarter of the time spent in parks by the elderly is in barrier-free open spaces^52^. Therefore, for an open park, the entrance and exit methods should be adjusted, steps should be reduced, and ramps should be set up at the main entrance for the disabled or the elderly to meet the needs of different groups of people.

### Limitations and future research

The goal of this research is to evaluate urban parks through questionnaire surveys and to investigate tourists’ satisfaction with infrastructure and the role of urban parks in promoting residents’ health^51^. There are several limitations in the research. First, this research is based on the evaluation of a single case and targeted optimization strategies made after the evaluation, which is not universally applicable. Second, the study was conducted in a large town park in China during the epidemic, and the results are not generalizable to other countries or developed regions. Third, due to the low cultural level of urban residents, the expression and description of the scene are not sufficient, as the respondents tended to provide vague evaluations of their own needs and satisfaction. Finally, the evaluation index system of this study is not sufficiently comprehensive; in particular, the regional influence of economic factors is not comprehensively investigated. The sample of the study is also quite small (N = 292). The composition of the sample means that it may be extended to parks and green spaces in similar impoverished areas^39^. However, the extent to which it can be extended to more diverse regions is limited.

The current research is the first step towards understanding how social groups of different ages in urban areas experience a single park. Other research will supplement these findings and address the limitations of the research^54^. For example, future studies may include more urban parks, make comprehensive assessments and comparisons, and analyze more samples to determine the needs and comfort improvement criteria for urban comprehensive parks, beautify the environment, improve the health index of the environment, and make parks more conducive to promoting the health and well-being of urban residents.

## Conclusion

In order to make parks a healthy resource, green space planning should eliminate and avoid obstacles to use, such as insufficient lighting and sanitation facilities. To meet the needs of different user groups and usage times, park planning should be inclusive and allow equal consideration of the usage preferences of residents in different regions^55^. Therefore, the specific local environment and social and cultural conditions, changing environment and climate adaptation must be considered in the park design process.

Through POE research and analysis of Qingkou Central Park, it was seen that small towns were mostly located in the fringe area of the city, with few venues for activities and a lack of entertainment facilities^56^. With the development of society and transportation, communication between small towns and cities is becoming increasingly closer. Residents in small towns aspire to have infrastructure similar to that of cities. As green public spaces in towns, comprehensive parks in small towns attract an increasing number of small-town residents. In the current transformation of new urban industrialization, residents in small towns are becoming younger, and with the liberalization of the two-child policy and the intensification of the aging trend, the number of urban infants and elderly is directly related to the use of parks and green spaces in small towns. Residents in small towns are paying increasing attention to the infrastructure convenience, functional diversity and cultural atmosphere of the park. Paying attention to the construction and use of small-town parks, on the one hand, will help promote the humanization of small town parks and beautify the urban environment; on the other hand, it will help maintain ecological balance, improve the health of small town residents, and make small town workers feel a sense of belonging. It will also increase the happiness index of residents in small towns.

The study found that tourists’ evaluation of comprehensive parks in small towns mainly depended on the following factors: functional facilities, regional humanities, park management, etc. First, there is a general problem of a low utilization rate in small town parks, and the construction of functional facilities can quickly and effectively improve the utilization rate of resources and the redistribution of land resources, increase public satisfaction, and form a virtuous circle of public participation mechanisms. Comprehensive parks in small towns have the advantages of being located in wide and large areas. Therefore, in addition to moving closer to urban parks in the setting of functional facilities, we should consider the needs of different age groups for parks and green spaces to meet the needs of more people. Second, as an important part of small towns, comprehensive parks need to inherit and develop the regional cultural characteristics of the town^57^. While enjoying green ecology and increasing cultural attributes, local cultural activities can be appropriately introduced. Cultural exhibitions should be held continuously to enhance the younger generation’s understanding of local culture and promote the spread of traditional culture. Third, comprehensive parks in small towns should introduce professionals to perform professional operations and practices on park facilities, equipment, and plant maintenance^58^. In addition, the elements of smart parks should be appropriately introduced into town parks, and some functional areas (such as badminton courts and basketball courts) can begin operating in a tidal mode. It is easier to increase public participation and increase the vitality of the park by adopting paid usage during periods of heavy traffic and adopting the free mode at noon or night hours with low traffic. Through reasonable planning, the income generated by operation will continue to be invested in the construction of the park, and the park environment and the construction and development of the park will be introduced into the ecological development model. The results of this study provide evidence for the natural environmental inequality faced by urban residents in China, but in the context of healthy cities, these inequalities cannot be reduced only by promoting and optimizing parks and green spaces^59^. We suggest that local governments in China implement more effective and sustainable park planning based on different situations and encourage public participation to achieve a true balance between the supply and demand of parks and green spaces in order to develop healthy cities.

## Data Availability

SPSS (version 25), AHP and GIS (version 10.5) were used for data analysis and visualization. All datasets and software used in the analysis are listed in Tables 2 and 3, Fig. 2.
